# Development of Therapeutic Monoclonal Antibodies for Emerging Arbovirus Infections

**DOI:** 10.3390/v15112177

**Published:** 2023-10-30

**Authors:** Leonardo F. Ormundo, Carolina T. Barreto, Lilian R. Tsuruta

**Affiliations:** 1 Biopharmaceuticals Laboratory, Instituto Butantan, São Paulo 05503-900, Brazil; leonardofontoura@usp.br (L.F.O.); carolinatbarreto@usp.br (C.T.B.); 2 The Interunits Graduate Program in Biotechnology, University of São Paulo, São Paulo 05503-900, Brazil

**Keywords:** arbovirus, monoclonal antibody, flavivirus, alphavirus, neutralizing antibody, antibody discovery

## Abstract

Antibody-based passive immunotherapy has been used effectively in the treatment and prophylaxis of infectious diseases. Outbreaks of emerging viral infections from arthropod-borne viruses (arboviruses) represent a global public health problem due to their rapid spread, urging measures and the treatment of infected individuals to combat them. Preparedness in advances in developing antivirals and relevant epidemiological studies protect us from damage and losses. Immunotherapy based on monoclonal antibodies (mAbs) has been shown to be very specific in combating infectious diseases and various other illnesses. Recent advances in mAb discovery techniques have allowed the development and approval of a wide number of therapeutic mAbs. This review focuses on the technological approaches available to select neutralizing mAbs for emerging arbovirus infections and the next-generation strategies to obtain highly effective and potent mAbs. The characteristics of mAbs developed as prophylactic and therapeutic antiviral agents for dengue, Zika, chikungunya, West Nile and tick-borne encephalitis virus are presented, as well as the protective effect demonstrated in animal model studies.

## 1. Introduction

The passive immunotherapy approach based on the introduction of antibodies was developed by Emil von Behring and Shibasaburo Kitasato in the late nineteenth century using serum to protect people against infectious diseases such as diphtheria and tetanus [1]. This approach was effective for several infectious diseases, but its use was reduced by antimicrobial drug approvals, and it was restricted to treating venom intoxication and some viral infections [2]. The technological revolution in discovery strategies to obtain monoclonal antibodies (mAbs), followed by microbial resistance to certain drugs, opened the opportunity to develop passive immunotherapy for a wide variety of infectious diseases without drug approval for prophylaxis and therapeutics [2], although few mAbs targeting infectious diseases have been approved to date [3]. Until now, there have been mAbs targeting only 3 viruses, respiratory syncytial virus (RSV), human immunodeficiency virus (HIV-1) and Ebola virus (EBOV), considering only viral infections [3]. The development of mAb therapy was initially directed at cancer and immunological diseases such as autoimmune disorders because of the high incidence of these conditions and the unavailability of effective drugs for treatment [2].

Recently, the pandemic of severe acute respiratory syndrome coronavirus 2 infections and COVID-19 disease changed this scenario because of the rapid spread of this infection and the urgent need to accelerate the development of drugs and vaccines to deal with the pandemic. The efforts from the scientific community were intensive and vaccines and antiviral drugs, including neutralizing mAbs, received emergency use authorization from the US Food and Drug Administration (FDA) in record time [3].

Infectious diseases caused by arthropod-borne viruses (arboviruses) are a challenging public health problem in tropical and subtropical developing countries that need a dynamic state of emergency because of potential outbreaks [4]. This is considered a global medical concern since emerging diseases do not impact only the population of these countries as a result of the intensive circulation of people because of globalization and tourism [5]. Furthermore, the spread of (re)emerging diseases is rapid when this happens, and thus, it is important to have a continuous effort and support for studies on the development of drugs and vaccines to control outbreaks.

In recent centuries, both hemispheres reported several cases of human emerging diseases caused by arboviruses, such as yellow fever virus (YFV), dengue virus (DENV), West Nile virus (WNV), chikungunya virus (CHIKV) and Zika virus (ZIKV) [5]. Highly effective vaccines for some arboviruses have been approved such as YFV [6], Japanese encephalitis virus (JEV) [7], tick-borne encephalitis virus (TBEV) [8] and tetravalent DENV [9], and others are under development [4]. However, antivirals or other therapeutics are not available for clinical use, and the development of specific therapeutics such as mAbs is under way. Passive immunotherapy using neutralizing mAbs in animal model-based studies protects against infection by genera *Flavivirus* [10] and *Alphavirus* [11] using specific mAbs. It was observed that mAbs with poor neutralizing activity can also provide protection in animals [11]. The biggest challenge is to demonstrate protection and efficacy in human therapy.

This review focuses on the mAb-based therapeutics for infectious diseases caused by arboviruses, specifically *Aedes*-borne RNA arboviruses DENV, ZIKV and CHIKV, and also WNV, which is a *Culex*-borne RNA arbovirus. mAbs for TBEV will also be presented. The mAb discovery technologies and improved strategies for mAb development are highlighted. First, we present an overview of mAb isolation technologies, and then neutralizing mAbs that have been developed to arboviruses. Finally, next-generation approaches to obtain mAbs with high efficacy and potency will be shown.

## 2. Technologies for mAb Discovery

### 2.1. Hybridoma Technology

**Figure 1:**
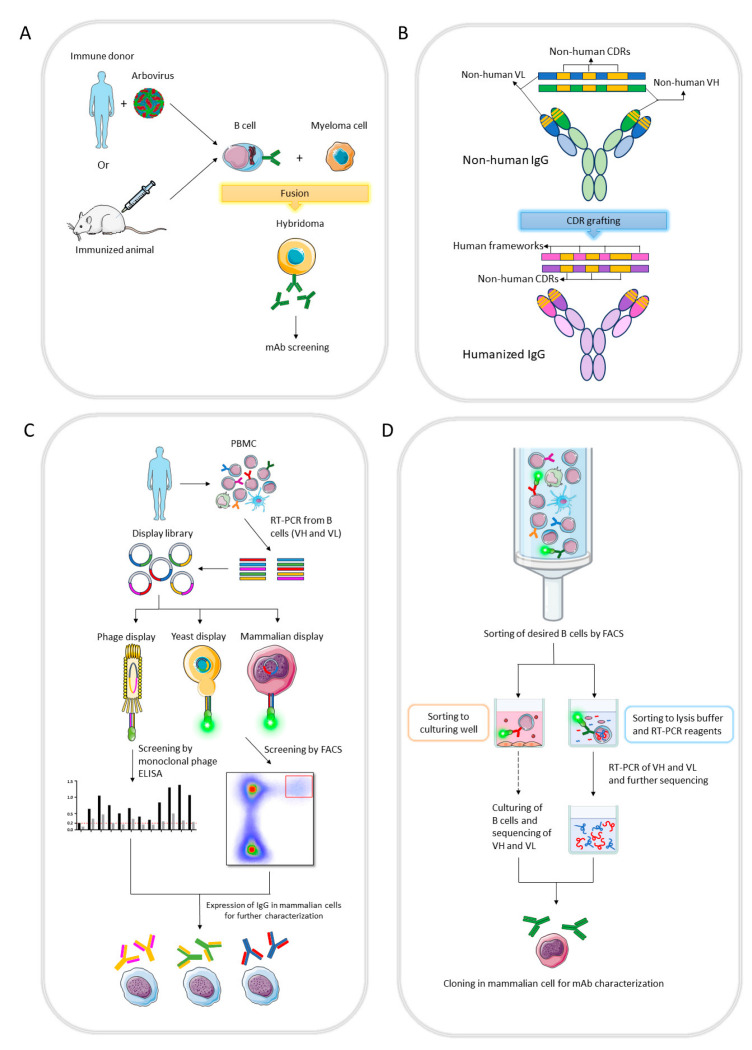
Technologies for mAb discovery. (**A**) Hybridoma. (**B**) Antibody humanization. (**C**) In vitro display technology. (**D**) B-cell sorting technology.

The first technology of mAb production was hybridoma culture developed by Köhler and Milstein in 1975 [12]. Hybridoma technology involves fusing antibody-producing B cells from an immunized animal, mainly mice, with murine myeloma cells resulting in hybrid cells that can produce unlimited quantities of a specific antibody (Figure 1A). The first therapeutic mAb, Orthoclone OKT3, was approved in 1986 [13]. However, clinical trials with hybridoma-derived mAbs were largely unsuccessful because murine mAbs were recognized as heterologous proteins and were highly immunogenic, generating human anti-mouse antibodies (HAMA) that affected the safety and therapeutic efficacy of the antibody [14,15].

The mAbs obtained from hybridoma culture retain the natural gene pairing of variable and constant regions, ensuring their proper functioning. Besides, this technology relies on B cells that were matured in secondary lymphatic organs in response to an antigen [16].

Human hybridoma produces human mAbs by the fusion of human B cells and fully human myeloma [17]. However, the application for therapeutic purposes has been limited because of several technical disadvantages. For example, high-efficacy human fusion partners are unavailable, the number of B cells is limited, fusion efficiency is low and production cost is high [17]. Heteromyeloma cells have been used to generate mAbs against viral diseases such as those caused by HIV, CHKV and DENV [16].

### 2.2. Immortalization of Human B Cells

The generation of a stable human B-cell line for mAb isolation and human B-cell repertoire study was challenging because of the limitation of maintaining B cells in vitro [18]. The immortalization of B cells by Epstein-Barr virus (EBV) transformation was developed to reach this objective [19] and involved four steps: B-cell isolation, EBV infection, B-cell cloning and screening strategies. However, the technique had low efficiency in relation to EBV infection, and B-cell cloning [20] limited the generation of large numbers of B cells. Improved methods increased the efficacy of the B-cell immortalization, and antibodies against SARS-CoV were identified [21]. B cells were also immortalized by transducing memory B cells with transcriptional factors, and a stable cell line was identified [22].

The immortalization of B cells has been combined with other techniques to obtain mAbs. One example is the generation of the human hybridoma-producing mAb for DENV by the fusion of EBV-transformed B cells with myeloma cells [23].

### 2.3. Antibody Humanization

Non-human mAbs administered in therapy may function as antigens and thereby elicit antibodies against them. To overcome this problem by reducing heterologous domains or residues in non-human mAbs, antibody humanization techniques were developed, replacing them with human domains or residues to minimize immunogenicity. The first technique was to obtain chimeric mAbs: antibody variable domains (light chain and heavy chain) from heterologous origin were combined with human constant domains [24]. The rational choice of constant domains is crucial to obtain chimeric mAbs. Although the variable domains are responsible for antigen binding, the constant regions may also play a role in this function [25]. Avidity is the strength of multiple interactions between antibodies and antigens to form a stable complex and is another important factor that interferes with binding properties. Chimeric mAbs can alter avidity through constant domain exchanges [26], and decreased binding could happen according to allosteric effects [27]. The isotype selection could also have an impact on chimeric mAb specificity [28]. Furthermore, these mAbs trigger immunogenicity responses generating human anti-chimeric antibodies (HACA) [29].

Techniques to obtain humanized antibodies were then developed to lower immunogenicity response. One promising technique is based on the transplantation of the highly specific sequence of antibodies called complementarity-determining regions (CDRs) of non-human origin on human framework sequences using molecular biology approaches, and it was designated CDR grafting [30] (Figure 1B). The first methodology cloned heterologous CDRs into human frameworks; however, this led to reduced affinity because of alterations in critical residues present in heterologous frameworks related to binding, and grafting did not preserve these features [31,32]. These residues in the frameworks that affect the conformation of CDR loops and contribute to the characteristics of mAbs are called Vernier zones [33]. The back mutation approach to revert these framework residues of humanized mAbs was used in the development of Zenapax^®^ (daclizumab), the FDA-approved mAb in 1997 for therapeutic use in patients with kidney transplantation [34]. All CDR grafting methodologies rely on the homology between the non-human mAb and human antibodies to decide the suitable human framework for humanization. Most humanized antibodies display reduced affinity toward antigens [35], and about 9% of them elicit human anti-humanized antibodies (HAHA) [29].

### 2.4. In Vitro Display Technology

In vitro display technology is the selection of DNA-binding proteins or antibodies based on the directed evolution approach [36]. In the field of antibody discovery, it mimics the in vivo process of antibody generation and has four main characteristics: (1) the generation of the genotypic diversity by library construction, (2) the linkage between the genotype and the phenotype, (3) the recovery of the DNA sequence encoding the selected antibody and finally (4) the antibody amplification. The isolated clones can be expressed to be characterized and the best candidates are selected [14] (Figure 1C). Further characterizations can be performed by IgG production in mammalian cells. Antibody libraries can be derived from either non-immunized (*naïve* and synthetic) or immunized donors. *Naïve* libraries should have high diversity, but the selected clones may not have high affinity. On the other hand, libraries from immunized donors have a lower diversity with higher affinity candidates [37,38].

The main advantage of this technology is the capability to select antibodies against a wide range of targets and epitopes since the selection from a *naïve* or synthetic antibody repertoire does not rely on an in vivo immune response. Thus, the isolation of antibodies targeting even self-antigens and toxic, unstable, and non-immunogenic antigens is possible using the library from non-immunized donors [14].

Phage, yeast and mammalian cell surface displays are examples of in vitro display technologies to select antibody fragments such as the single-chain variable fragment (scFv), antigen-binding fragment (Fab) and single-domain antibody (sdAb) [37,38,39].

Phage display was the first technology developed to select exogenous peptides on the filamentous M13 bacteriophage (phage) surface with specific binding properties by Smith et al. in 1985 [40]. Antibody phage display was developed by McCafferty and colleagues five years later [41]. It is widely used in the discovery of therapeutic mAbs because of several advantages: low cost of the *E. coli* expression system [42], finding antibodies with desired properties using large *naïve* libraries [37], versatile selection process with the possibility of using diverse conditions [14], affinity maturation approach possible to improve binding properties [42], possibility to humanize antibody by guided selection technique [14], and a high level of customization and also full control of the experiment at each stage by direct and rational approach [42].

### 2.5. B-Cell Sorting Technology

After the revolutionary hybridoma technology and in vitro display technologies, single B-cell sorting was developed for mAb discovery. Antibody-secreting cells (ASC) and B cells have a very short lifespan ex vivo, and therefore, there is a need for the immortalization of these cells, i.e., by hybridoma technology that allows B-cell antibody screening through analyzing hybrid cells. Novel advances allowing cell culture of ASC and B cells were developed enabling the screening of a large number of mAbs [43,44,45]. Thus, to recover broadly neutralizing mAbs, an efficient screening approach of a larger number of mAbs is important, given that a minority of B cells have these features.

A well-known method to isolate single B cells is fluorescence-activated cell sorting (FACS). Surface markers, such as membrane-bound antibodies (B-cell receptor, BCR) in B cells are a suitable candidate to isolate cells with specific antibodies for a given antigen. The antigen of interest must be attached to a fluorophore and used as bait to sort the single B cells bearing antibodies. Cells can be sorted into wells for further culture for mAb screening and accessing genetic material [43,45] (Figure 1D) or even prolong their lifespan through EBV immortalization [46,47]. Alternatively, cells can be sorted into wells with reagents for high-throughput sequencing [48,49].

After the B-cell sorting step, the antibody screening step starts and demands high-throughput systems in function of a high number of candidates and multiple steps. Single-cell reverse transcription PCR (RT-PCR) is performed to identify the sequences of the heavy chain and light chain genes encoded by each antibody. The antibody genes are then cloned into an expression vector and transfected to the eukaryotic cell line to obtain mAb for testing binding and analyzing functional features [50,51]. The full process is long and work-intensive, yielding few promising and specific candidates.

While FACS is one of the most commonly used methods for mAb discovery from single B cells, there are limitations. Plasma cells, a differentiated stage of B cells that are highly specialized in secreting antibodies, do not possess a BCR. However, using FACS for B cells has significant advantages, such as a relatively low cost, simplicity, and a high throughput platform [51].

Modern technology for single B-cell investigation uses microfluidic platforms. Berkeley Lights Beacon^TM^ is an example of a highly efficient and automated microfluidic system that enables rapid interrogation of B cells in a high-throughput manner [52]. In this technology, B cells are sorted into individual channels using OptoElectro Positioning (OEP), each having a very small volume (usually ranging from 740 pL to 1.7 nL), with culture chips customized for specific needs [51,53,54]. The culture supernatant is further analyzed, and several parameters are assessed related to mAb and cell clone screening, such as IgG productivity, cell expansion rate, and surface markers [51,53,54]. It represents an example of an open system, where information about ASC can be readily accessed in real-time.

Another example of an open microfluidic system is based on microengraved polydimethylsiloxane (PDMS) chips. These chips contain nanowells into which cultured B cells are added and sorted [55]. After sorting, the ASCs with specific antibodies for a given antigen are analyzed in microarrays, and genetic material can be recovered for further analyses [55]. Although this methodology enables high-throughput analyses, it does not provide the same level of information as the Berkeley Lights Beacon^TM^, albeit a less expensive technology, making it more accessible.

Closed microfluidic systems, also known as closed systems, are based on encapsulating cells in droplets embedded in an oil emulsion. They are referred to as closed systems due to the need to break the encapsulated droplet to access the information inside the reaction. The sequencing of individual B cells may be obtained with the cDNA synthesized inside the droplets [56,57]. Other closed microfluidic methodologies do not sort B cells prior to microfluidic encapsulation; instead, they rely on B-cell identification with specific antibodies for antigens of interest within the droplets, and material for sequencing is also obtained within the droplets [58].

The greatest advantage of microfluidic systems is that they operate with very small volumes, allowing for minimal reagent use, rapid screening of mAbs, and quick achievement of the molar amount of antibody needed for identifying ligands and other characteristics [51]. Additionally, they can also be used for high-throughput studies [51]. However, these systems do not currently support functional analyses, such as neutralization assays, an important characteristic to be assessed for therapeutic mAbs, and therefore, these assays should be performed after mAb isolation [51].

The primary advantage of B cell-based technologies for clinical purposes is the preservation of the native pairing of the variable domains of the B cell. Antibodies undergo affinity maturation in vivo, and those with auto-reactivity undergo clonal deletion [59]. This mechanism helps minimize the selection of potential autoreactive and non-specific mAbs [51]. Native-paired mAbs may also exhibit lower immunogenicity and have greater developability, a common problem faced by mAbs obtained from libraries that do not retain native pairing [51].

## 3. Development of the Therapeutic mAbs Targeting Arboviruses

### 3.1. Characterization of Arboviruses

Arboviruses are a diverse group of viruses whose vectors are arthropods, including different families such as *Flaviviridae* and *Togaviridae,* among others. These two families have worldwide medical importance due to representing human viral pathogens causing several emerging diseases [60].

**Figure 2:**
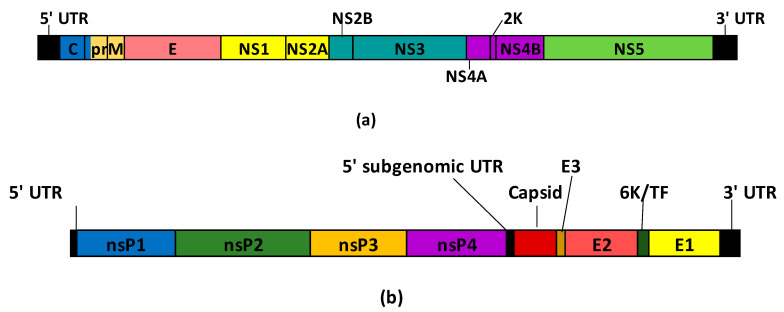
Schematic representation of the polypeptides encoded by flavivirus (**a**) and alphavirus (**b**) genomes adapted from Pierson and Diamond 2020 [61] and Rupp et al. [65], respectively. (**a**) Flavivirus genome has a single open reading frame (ORF) and encodes three structural proteins, capsid (C), pre-membrane (pr) and envelope (E), followed by seven nonstructural proteins (NS1, NS2A, NS2B, NS3, NS4A, NS4B and NS5). 2K: domain between NS4A and NS4B. (**b**) Alphavirus genome has two ORFs. The first ORF translates four nonstructural proteins (nsP1, nsP2, nsP3 and nsP4) from genomic RNA, while the second ORF encodes five structural proteins (capsid, E3, E2, 6K/TF and E1) through a subgenomic mRNA. 5′ subgenomic UTR: a third UTR between two ORFs.

Flaviviruses are spherical particles containing positive-sense RNA that encodes three structural proteins, such as pre-membrane (prM), envelope (E) and capsid (C), and seven nonstructural proteins (NS1, NS2A, NS2B, NS3, NS4A, NS4B and NS5) [10,61] (Figure 2a). The E protein forms a dimeric structure anchoring on the M protein, mediates virus entry and has three domains: DI, DII and DIII [61]. Flavivirus-infected individuals present neutralizing antibodies to E, prM and NS1 proteins [62,63]. DENV, ZIKV, WNV and TBEV are examples of flaviviruses, and therapeutic mAbs developed for them will be presented in the review.

Alphaviruses are positive-sense RNA viruses of the family *Togaviridae* with an icosahedral nucleocapsid encoding four nonstructural proteins (nsP1, nsP2, nsP3 and nsP4) and five structural proteins (capsid, E3, E2, 6K/TF and E1) [11] (Figure 2b). Host humoral antibodies have as target E1 and E2 proteins [11,64]. CHIKV is an alphavirus and mAbs for it will be considered.

The vector species, such as mosquitoes or ticks, responsible for flavivirus diseases primarily define the epidemiology [10]. The clinical manifestation that differentiates the diseases caused by two types of mosquitoes is that *Aedes*-borne viruses are characterized mainly by fever, influenza-like symptoms and/or hemorrhagic illness, whereas *Culex*-borne viruses such as WNV are encephalitic infections [5].

### 3.2. Dengue Virus (DENV)

Dengue infection is one of the most common mosquito-borne diseases in tropical countries [66]. The dengue virus has four serotypes: DENV1, DENV2 DENV3 and DENV4, and all of them can cause asymptomatic to severe cases, including hemorrhagic fever [67]. DENV1 is the most prevalent DENV serotype. The infection can elicit long-lasting memory B cells, but serotype cross-reactive antibodies can bind or cause weak DENV neutralization, leading to virus entrance into cells via FcɣR and causing antibody-dependent enhancement (ADE) [23,68]. Besides, non-specific T memory cells lead to cytokine storm and poor outcomes in the second infection, a phenomenon known as original antigenic sin [69]. These two immunological aspects are an impairment in the development of vaccines for DENV. There are also two mAbs for dengue treatment in clinical trials, both in phase I [70,71].

There is evidence that the E protein mediates virus entry, and it is proposed that DIII of the E protein is involved in entry into host cells since a virus with mutated DIII was unable to enter cells [72,73]. Because of the diversity among DENV serotypes, some mAbs may not be neutralized by others [10].

MAb-based therapies have certain advantages in dengue treatment since most of the antibodies elicited by the infection are non-neutralizing with the ADE risk that comes with polyclonal antibodies. Recombinant mAbs are promising drugs as they are not likely to present adverse events. As the ADE phenomenon is more likely to occur with serotype-specific antibodies that just cross-react with other serotypes [74], we focused on the DENV antibodies that cross-neutralize different serotypes (Table 1).

#### MAbs for DENV

The development of mAbs targeting specifically the DENV1 serotype is urgent since it causes predominantly infection. Mice were immunized with UV-inactivated DENV1 particles and recombinant DENV1 E protein, and hybridomas producing DV1-E1 and DV1-E2 mAbs were isolated. The epitope was characterized as DIII of the E protein, in the region between residues Gln347 and Asp360, which plays an important role in DENV1 neutralization, and it is not a conserved region among other serotypes [82].

The 4E11 Fab was obtained by hybridoma technology from mice immunized with DENV4 and has as a target the DIII of the E protein. It neutralized all four DENV serotypes with different potency [75]. The IgG2a format of this mAb neutralized DENV with greater potency than did the Fab, indicating that the in vivo properties of the mAb are desirable for therapeutic purposes [75].

MAbs were generated by the fusion of the PBMC from DENV2 acute-phase or DENV-infected convalescent donors with SPYMEG cells and revealed that mAbs derived from acute-phase patients had cross-reactivity and neutralization capacity with all four serotypes [83].

In another approach, mAbs to DENV E protein were identified from two patients during acute-phase secondary infection by single plasmablast sorting [84]. These mAbs displayed strong neutralization activity against multiple serotypes in vitro and showed higher protection of the previous serotype infection because of the antigenic sin phenomenon [84]. Further studies selected the SIgN-3C mAb, which binds to a quaternary epitope in the inter-dimer interface of the E protein, specifically to the DII fusion loop and DIII [79]. The epitope of this antibody is extensive, giving the antibody the capacity to cover the inter-dimer region because of the long CDR3 sequence [79]. SIgN-3C has neutralizing capacity across all four DENV serotypes.

Neutralizing mAbs were also isolated by DENV2 E envelope-specific memory B cells from individuals recovered from natural infections, and some mAbs presented neutralization potency to more than one serotype [45]. Memory B cells represent a very small part of PBMC, needing to choose the right period for mAb selection.

Discovery of mAbs targeting diverse epitopes helps the design of effective therapeutic drugs. d448 is a broadly DENV-neutralizing mAb, obtained by B-cell sorting of Rhesus macaques immunized with dengue vaccine, and it binds to DII at the interface of E and M proteins; Li et al. were the first to report on this epitope [81]. This mAb has cross-reactivity and cross-neutralization potency with four DENV serotypes [81].

The fusion loop of the E protein is the epitope for weak neutralizing antibodies [78], but potent antibodies targeting this epitope have been selected. The 3G9 mAb is one example obtained by a hybridoma generated from a patient in the acute phase of a secondary DENV infection, and the variable region showed high somatic hypermutation rates [78]. This mAb neutralized in vitro all four serotypes and also JEV, WNV and ZIKV. Besides, 3G9 displayed in vivo protection and therapeutic potential [78].

Another mAb targeting the fusion loop is 2A10G6, which binds to a new epitope, 98DRXW101 motif, highly conserved among different flaviviruses [76]. This antibody was obtained by a hybridoma using B cells of mice immunized with DENV2 [76]. 2A10G6 showed protection against lethal infection caused by DENV1-4 when administered at the same time as the viral challenge in a suckling model. The effects of 2A10G6 were dose-dependent and gave full protection against DENV2 and partial protection against DENV1, 3, and 4 [76].

A panel of mAbs targeting a new epitope called envelope dimer epitope (EDE) that connects two E protein subunits were identified by plasmablast sorting from DENV secondary infected patients with hemorrhagic fever symptoms. These mAbs are broadly reactive and capable of neutralizing multiple DENV serotypes [85].

NS1 is another DENV protein used to trigger antibodies and can protect mice against infection. NS1 has molecular mimicry with proteins of the mammalian host, and a modified peptide for the NS1 wing domain region not recognized by the host was synthesized for the immunization of mice [86]. 2E8 and 33D2 mAbs were obtained by hybridoma cells and recognized all DENV serotypes [86]. These mAbs were humanized by CDR grafting, and in vivo, the therapeutic potential was evaluated in a mouse model, where both mAbs caused a decrease in DENV-induced prolonged bleeding time and skin hemorrhage, while the humanized mAb, h33D2, also decreased viremia [87].

### 3.3. Zika Virus (ZIKV)

Zika disease caused by ZIKV is transmitted by *Aedes* spp. mosquitoes [88] and also sexually [89]. While asymptomatic infections account for around 80% of cases, Zika symptoms include rash, fever, and headaches, with Guillain-Barré syndrome, which can be a sequala [90]. Importantly, ZIKV shows tropism to immune-privileged sites [91], such as the brain [92] and the placenta [93]. This tropism to the placenta and the ZIKV vertical transmission can lead to abnormalities in brain development [94,95] and even spontaneous abortion [95]. Microcephaly is a common morphological abnormality, as well as brain calcification [94,95]. However, even without apparent morphological disorders in brain development, congenital zika may lead to neurodevelopment impairments in children [96]. Although there is no FDA-approved vaccine for ZIKV, there is currently one mAb for Zika disease treatment in phase I clinical trial [97].

ZIKV and DENV E proteins show similarity of around 56%, and consequently, antibodies triggered by ZIKV infection are widely cross-reactive with DENV [98]. Similarly to DENV, the depletion of B cells reactive to the soluble ZIKV E protein does not significantly affect the neutralization of ZIKV immune sera, suggesting that the quaternary epitopes, i.e., those present in structural virion arrangement, may be responsible for the neutralizing response [99].

Plasmablasts from ZIKV-infected patients with previous dengue episodes were shown to have clonal expansion and high rates of somatic hypermutation due to a secondary infection, differing from plasmablasts derived from DENV-naïve ZIKV-infected patients. ZIKV-neutralizing antibody from dengue experienced-donors developed DENV cross-reactivity response [100]. In another study, higher levels of ZIKV- ZIKV-neutralizing antibodies after ZIKV exposure were associated with their reactivity to DENV1 DIII of the E protein [101].

Antibodies against DENV, exhibiting cross-reactivity with ZIKV at suboptimal concentrations or with poor neutralization potency led to ADE in vitro models [102] and also in mouse models [103]. Since ZIKV circulates in DENV endemic areas, the ADE phenomenon happens during infections among these viruses, and it is of fundamental importance to investigate this characteristic in therapeutic antibodies. The effects of ZIKV in fetal development are also relevant when therapeutic effects are evaluated and when gestational models should be considered.

#### MAbs for Zika Disease

Therapeutic mAbs developed for Zika disease with neutralizing activity for ZIKV are summarized in Table 2.

A panel of human DENV mAbs that binds to E protein was identified and presented in the DENV section [85]. Some potent and neutralizing DENV mAbs targeting envelope dimer epitope (EDE) [85] showed cross-reactivity with ZIKV and inhibited ADE of ZIKV infection [102]. EDE1-B10 has therapeutic potential against DENV and ZIKV since it inhibited virus strains and protected ZIKV-infected mice against lethality. Besides reducing viral persistence in immune-privileged tissues, such as the brain and testicles, this mAb protects animals against tissue injury and virus transmission to the fetus [110]. In a rhesus monkey study, EDE1-B10 showed therapeutic and prophylactic efficacy against ZIKV [109].

SIgN-3C is another DENV mAb binding to a quaternary epitope, mentioned in the DENV section, that showed in vivo efficacy against ZIKV [110]. In non-pregnant mice, mAb administration exhibited therapeutic effects against lethal infection, viremia and weight loss [110]. In pregnant mice infected on embryonic day 10.5, mAb administration resulted in normal fetal weight and reduced viral load in amniotic fluid and organs, showing potential therapeutic and prophylactic effects [110].

ZIKV-117 is a representative potent mAb against ZIKV that binds to a quaternary epitope in the inter-dimer interface of protein E. It was obtained by hybridoma fusing B cells from a convalescent patient who was selected after a deeper serum response investigation [104]. ZIKV-117 neutralized ZIKV strains and failed to neutralize DENV, all serotypes. When administered to mice, ZIKV-117 reduced Zika lethality; in the prophylactic approach, post-exposure therapy in mouse dams showed a significant viral burden decrease in the placenta, maternal tissues, serum and fetus tissues with also a reduction in fetal demise [104]. Besides, no mutant escapes were detected after six passages in the presence of ZIKV-117 [104]. ZIKV-117 was formulated as lipid nanostructures with the mRNA encoding the mAb, and robust protection was shown in non-pregnant mice [105].

Z23 and Z3L1 mAbs were isolated from memory B cells sorted from a convalescent Zika patient and recognized different tertiary epitopes in the E protein [107]. Z23 binding site is at the top of DIII, while Z3L1 binds to an epitope ranging from DI to DII [107]. These mAbs strongly neutralized ZIKV with no cross-reactivity with any of the four DENV serotypes, and mice treated with each mAb were completely protected against ZIKV infection without weight loss [107].

Anti-NS1 antibodies represent an alternative approach to protect against ZIKV. A panel of ZIKV murine and human mAbs targeting NS1 was identified through hybridomas secreting murine mAbs [111] or B-cell sorting of ZIKV-infected donors presented previously [104]. 749-A4 mAb was isolated by plasmablast sorting from a dengue patient, showing cross-reactivity with ZIKV NS1 [111]. Murine Z17, human ZIKV-292 and human 794-A4 mAbs recognized NS1 residues in the C-terminal region of the β-platform and gave protection in immunocompromised mice receiving lethal ZIKV challenge, and the viral RNA level was reduced in the fetuses of pregnant mice [111].

An approach to avoid escape mutants in ZIKV is the administration of a combination of two or more mAbs targeting distinct epitopes. Z004 mAb was selected from memory B-cell sorting of ZIKV-infected patients, showing reactivity to ZIKV and DENV-1, and targeting the lateral ridge of DIII of the E protein with potent neutralizing activity in vitro. It also protected mice and decreased symptoms and lethality when given pre- and post-infection [101]. High-dose ZIKV challenge in rhesus macaques was tested and showed prolonged viremia which led to escape virus mutants. Z021 mAb was isolated with the same B-cell sorting strategy as Z004, with similar characteristics of this mAb related to reactivity and neutralizing potency in vitro and in vivo; however, Z021 recognizes EDIII nearby or overlapping epitope by Z004 [106]. When Z004 and Z021 were administered as a cocktail in monkeys before receiving the high-dose ZIKV challenge, infection was delayed and viremia was reduced, preventing ZIKV escape mutants [106].

Therapeutic antibodies are usually IgG, but memory B-cell sorting of a pregnant patient with secondary ZIKV infection yielded ultrapotent DH1017. IgM, targeting a quaternary epitope in the E dimer in DII [46]. All variable domains of DH1017.IgM could bind to the same virion, but neutralization seems to occur because of viral aggregation [46]. The neutralization of this mAb is dependent on isotype since IgG was expressed and did not neutralize ZIKV and induce ADE, and besides, IgM protected mice more effectively from the infection, with reduced viremia [46].

The development of new strategies to accelerate therapeutic mAb discovery should be relevant since the selection of potential candidates is a long process. An integrated workflow that combined the discovery and the validation of the protective efficacy in animals was designated to identify promising ZIKV mAbs in a shorter time. High-throughput investigation of B cells from immune donors can rapidly provide a large number of mAbs, and this strategy could be applied for a rapid response in case a new disease outbreak emerges in the future [56].

### 3.4. Chikungunya Virus (CHIKV)

CHIKV is an alphavirus associated with musculoskeletal disease (arthritogenic alphaviruses), including Mayaro virus (MAYV), Ross River virus (RRV), O’nyong-nyong virus (ONNV) and Semliki Forest virus (SFV). These viruses are globally distributed [112] and the infection causes a severe and debilitating illness. The most common symptom is fever, but infection can also provoke joint pain, rash, and headache. In some cases, the joint pain can be so severe that it can last for months or even years [113,114,115].

The genotypes Asian, East/Central/South African (ECSA) and West African (WA) are known and CHIKV has limited genetic diversity among strains with 95.2 to 98% amino acid identity [116]. Antibodies raised against one strain can react with all others, leading to the broad consensus that CHIKV lineages constitute a single serotype [114,117,118,119].

Antivirals for prophylaxis or therapy have not been approved yet, and current treatment options are purely symptomatic. Development of mAbs against CHIKV has been under way (Table 3) and could be an alternative for clinical treatment.

#### mAbs for CHIKV

CHK-152 and CHK-166 are murine mAbs that target CHIKV E2 and E1 proteins, respectively, and were selected from a panel of CHIKV-neutralizing mAbs produced by hybridomas using mice immunized with CHIKV virus-like particles (VLPs) [114]. A single dose of the combination of two mAbs was effective in limiting the development of resistance to the antibodies and protected immunocompromised mice from the disease when given 24 to 36 h before CHIKV-induced death in post-exposure therapeutic trials [114]. Rhesus macaques received the mAb combination and it was effective in reducing viral spread and infection at distant tissue sites; however, residual viral RNA was present in tissues and organs and additional treatments could be needed to fully eliminate CHIKV [120].

The E2 protein is a major target for mAbs, and some mAbs were thus developed: 4N12 [115], SVIR023 [121] and DC2.271B [116].

4N12 is a fully human IgG2 kappa mAb that neutralizes in vitro three genotypes of CHIKV and was selected from a single individual who had CHIKV infection in Sri Lanka in 2006. A stable hybridoma was generated, and 4N12 neutralized the wild-type virus, as well as mutant forms. 4N12 was able to protect mice against CHIKV-induced death, even when administered after infection [115]. 4N12 mAb was then improved by moving CDR sequences to another framework, resulting in SVIR001 mAb, displaying similar antigen binding and neutralizing activity as the parental mAb. This modification was made to address some of the challenges that 4N12 mAb faced, such as the limited ability to control acute infection, the inability to reduce viral persistence, and the potential to cause long-term joint disease. SVIR001 was administered to CHIKV-infected Rhesus macaques and showed reduced viremia and the potential to decrease CHIKV-associated inflammatory diseases [122].

SVIR023 is a human IgG1 mAb derived from a human hybridoma that showed neutralizing activity against three clades of CHIKV. When administered until three days post-infection, it reduced virus number in various tissues. It was effective in preventing disease in mice when administered up to 4 weeks prior to the virus challenge. CHIKV-infected mice treated with mAb were resistant to the secondary challenge, and no evidence of ADE was detected [121].

DC2.271B is a human IgG1 mAb that binds the CHIKV E2 protein between the β-connector region and the B domain. Single B-cell sorting was used to isolate human mAbs from CHIKV-infected convalescent donors. The lethal viral dose challenge in mice showed that this mAb was highly protective after prophylactic and therapeutic administration [116].

An immune library was constructed with CHIKV-infected individuals and IM-CKV063 was selected by phage display technology using structurally intact E1/E2 on VLPs as a target. This mAb showed high neutralizing activity, and therapeutic and prophylactic protection in multiple-animal models up to 24 h post-exposure was observed [123].

Single-domain antibodies (sdAbs) are alternatives to conventional antibodies for diagnostic and therapeutic applications. CC3 and CA6 are nanobodies, obtained by llamas immunized with CHIKV VLPs, they bind to both the VLPs and the recombinant E1 protein and have neutralizing activity [124].

Broadly neutralizing antibodies that protect against several arthritogenic alphaviruses should be an interesting therapeutic approach. DC2.M16 and DC2.M357 are examples of human mAbs isolated from a donor previously exposed to CHIKV by a single B-cell sorting using MAYV E3-E2-E1 recombinant protein as a target. They showed neutralizing activity against CHIKV and MAYV, and DC2.M357 also neutralized RRV, ONNV and SFV. Both mAbs protected mice from CHIKV- and MAYV-induced musculoskeletal disease [112].

Pan-protective mAb refers to mAbs that can protect against more than one disease [113,114,115] and provides another relevant therapeutic approach. DC2.112 and DC2.315 are pan-protective and weakly neutralizing human mAbs that bind to a conserved epitope in alphaviruses in the DII of the E1 protein, located near the fusion peptide. They were selected by B-cell sorting from CHIKV seropositive individuals, bind to a variety of alphaviruses, those causing arthritis (CHIKV and MAYV) and encephalitis (Venezuelan, Eastern and Western equine encephalitis). The passive transfer approach of each mAb was tested in mice to evaluate the protection. Mice were protected from musculoskeletal disease induced by CHIKV and MAYV and the lethal neurological infectious disease provoked by encephalitis viruses [125].

### 3.5. West Nile Virus (WNV)

WNV emerged as an important cause of viral encephalitis and is maintained between mosquito vectors and birds in an enzootic cycle. However, it can cause infection disease in humans, horses and other vertebrate animals [126,127]. In humans, the infection is characterized by febrile illness that could advance to meningitis, encephalitis, and even fatal disease, especially for elderly and immunocompromised individuals [128].

Nine evolutionary lineages of WNV have been identified, and only strains of lineage 1 and 2 (WNV-1 and WNV-2) were responsible for human infections [129]. WNV-1 was described in Africa, Europe, the Middle East, Australasia and India, while WNV-2 showed less virulence, occurring in Sub-Saharan Africa, Madagascar and Europe [130]. WNV disease outbreak was in the Middle East, Europe and Africa and then spread to North America and other Americas [126].

#### mAbs for WNV

Table 4 presents mAbs that have been developed for WNV infection.

E16 mAb, derived from hybridoma, recognized WNV DIII of the E protein and had in vitro and in vivo inhibitory potency. Hm-E16 or hE16 is the humanized mAb version, obtained by the CDR grafting technique, that presented similar affinity and efficacy when mice were administered post-exposure [131,132]. Studies in WNV-infected hamsters showed that, when hE16 was administered after the virus reached the neurons in the brain, the treatment improved survival [133] and ameliorated neurological disease after viral replication [134]. Then the humanized mAb was designated as MGAWN1 to enter phase 1 clinical study [136]. The phase 1 clinical trial was registered with NCT00515385 and was completed in 2009, confirming the safety of a single intravenous infusion of up to 30 mg/kg of MGAWN1 in healthy adults [136,142]. The phase 2 trial (NCT00927953) was designed to study the WNV treatment with MGAWN1 and was terminated early due to the low enrollment [143].

Phage display scFv immune library was constructed with peripheral blood donated by three WNV-infected individuals and neutralizing mAbs targeting DIII of the E protein were selected [138]. CR4354 is a fully human IgG1 mAb derived from this immune library showing strong neutralizing activity against WNV I and protecting mice against lethal infection [139]. It neutralizes WNV infection at a post-attachment stage in the viral life cycle by blocking the pH-induced rearrangement of the E protein that prevents the virus fusion to the endosomal membrane [139,140]. Structural determination of the WNV-antibody complex was performed to determine the epitope and the neutralization mechanism was suggested as blocking virus fusion with the endosomal membrane [140].

Non-immune human phage display library was screened with WNV E protein and identified some scFvs. The scFv epitope was within the DI and DII sites of the E protein. The scFv-Fc were developed and five mAbs protected 100% of the mice from death when given prior to the virus infection [144].

MIT89 is a fully human IgG1 neutralizing mAb specific for WNV E protein, identified from WNV convalescent subjects, by combining the single B-cell sorting and the next-generation sequencing (NGS). Immune response study of the infection disease, using the integration of single-cell data, serum analysis and repertoire sequencing data, is a relevant approach that can be applied to other diseases [55].

WNV-86 is a neutralizing mAb to the WNV DII E protein that recognizes mature virions inhibiting the virus infection and dissemination. This contrasts with other flavivirus-specific antibodies, which tend to recognize immature virions and may rely on Fc-mediated functions to provide protection. Wild-type and L234A/L235A (LALA) mutant of WNV-86 significantly reduced viral burden in the spinal cord and brain of infected animals [137].

WN_83 is a human mAb that binds to WNV DIII of the E protein and was isolated from B cells from individuals vaccinated with inactivated JEV presenting WNV- and JEV-neutralizing antibodies in the sera. Recombinant WNV E protein was used to isolate WN_83 and it neutralized WNV both in vitro and in a WNV-inoculated mouse model [141].

### 3.6. Tick-Borne Encephalitis Virus (TBEV)

Tick-borne encephalitis (TBE) is a flavivirus infection that affects the central nervous system and is caused by TBEV. TBEV has three major subtypes: European, Siberian and Far-Eastern [145]. TBE is endemic in the “TBE belt” area comprising Central Europe, the Baltic region, Russia and part of eastern Asia [146]. The majority of human TBE cases are mild, but severe TBE can lead to sequelae and death [8].

Although ADE of TBEV infection was demonstrated in murine peritoneal macrophages in vitro [147], it was not detected in the in vivo model [148,149]. Passive administration of polyclonal antibodies for TBE treatment was discontinued in Europe because of possible ADE in two patients, but it still remains in use in Russia [149]. The development of neutralizing mAbs for TBEV is necessary as a better alternative [150].

#### mAbs for TBEV

The neutralizing mAb 14D5 was obtained by the hybridoma technology targeting DIII of the E protein of the Far-Eastern subtype [151,152]. Therapeutic and prophylactic efficacy was tested in BALB/c mice, and the prophylactic administration was more effective [152]. Chimeric mAb of 14D5 was constructed (ch14D5a) and displayed improved neutralizing potency than murine mAb [153]. Mouse protection was examined against TBEV lethal infection for both mAbs administered 24 h after virus exposure, and ch14D5a mAb provided a 100% survival rate, while 14D5 mAb achieved 70% [153]. A stable cell line producing chimeric mAb designed as chFVN145 was obtained and TBEV-infected mice receiving higher mAb dose had better protection. In the therapeutic approach, chFVN145 was given one, two and three days after infection of mice, and efficacy was found to be dependent on TBEV dose [154].

The murine mAb 13D6 targeting DIII of the E protein showed in vivo neutralization property, and Levanov et al. generated chimeric mAb (ch13D6) [155]. The affinity and neutralization activity of ch13D6 mAb was better than that of murine mAb [155].

Neutralizing human mAb T025 was isolated by single B-cell sorting from TBEV-infected convalescent individuals, and its epitope is the lateral ridge of DIII of the E protein [156]. T025 showed cross-reactivity and cross-neutralization with other tick-borne flaviviruses such as louping ill virus (LIV) and Omsk hemorrhagic fever virus (OHFV), besides binding to Kyasanur Forest disease virus (KFDV) and Langat virus (LGTV). In the prophylactic approach, BALB/c mice received T025 mAb 24 h before the TBEV challenge and they were protected. In the therapeutic scheme, T025 was administered after TBEV infection and early treatment was effective [156].

## 4. Next-Generation Strategies for Therapeutic mAbs

Earlier studies on mAb discovery targeting arboviruses selected non-human mAbs. However, advances in technological platforms, such as engineering techniques, have led to the isolation of human mAbs with characteristics for therapeutic purposes. Table 5 summarizes mAbs obtained by next-generation strategies to obtain improved mAbs.

One point that should be taken into consideration when developing therapeutic mAbs for infectious diseases, especially for an RNA virus such as arbovirus, is high mutation rate-generating variants. Therapeutic use of the neutralizing antibodies may also pose a selective pressure, obtaining escape mutants and leading to an ineffective treatment [60]. One approach to avoid this is the combination of various mAbs targeting different neutralization epitopes as mentioned in previous sections, which was applied to some therapeutic mAbs developed for arboviruses.

The ADE phenomenon is another point of attention when therapeutic mAbs are being developed for flaviviruses. ADE is well known with DENV antibodies in secondary infection, leading to hemorrhagic fever [23,68], but not only antibodies for the same virus may cause this phenomenon. It occurred for DENV antibodies used for Zika infection treatment in the in vitro model [102] and also in the murine model [103]. The main mechanism proposed for ADE is the more efficient infection of Fcɣ receptor-expressing myeloid cells in vivo [68]. This inconvenience can be addressed by the antibody engineering approach, focusing on the Fc portion. The L234A/L235A (LALA) and N297A Fc mutations are commonly used mutations that dramatically reduce the affinity of Fc for the Fcɣ receptor [163]. Many antibodies for arboviruses have the LALA mutation to abrogate the ADE phenomenon (Table 5). However, in some cases this engineering approach completely abolished Fc-dependent responses, i.e., effector functions, and therefore, careful evaluation is needed when this strategy is applied [164].

The application of both approaches, the mAb cocktail and LALA mutation could generate improved mAbs for therapeutic purposes. SMZAb1, SMZAb2 and SMZAb5 are mAbs to ZIKV derived by plasmablast sorting from ZIKV-infected subjects and exhibited therapeutic potential when used as a cocktail since each mAb binds an epitope overlapping the fusion loop of the E protein. SMZAb1 and SMZAb5 target DIII, while SMZAb2 binds to DII. Some cross-reactivity with DENV was observed in these three mAbs, and Fc LALA mutations were introduced to prevent potential ADE. The cocktail of engineered mAbs were administered to non-pregnant Rhesus monkeys one day before the ZIKV challenge, and viral replication was completely prevented in a prophylaxis regimen [161]. The same cocktail was administered to pregnant Rhesus macaques ZIKV-infected at peak viremia, and the treatment was effective in clearing the virus; however, viral RNA was present in amniotic fluid and failed to prevent fetal demise [165]. Thus, the treatment was not capable of stopping vertical transmission.

Antibody engineering is a promising approach to obtain improved therapeutic mAbs. VIS513 is a humanized IgG1 antibody for DENV obtained by antibody engineering of the murine 4E11 mAb, which neutralized all four DENV serotypes [75]. Initially, the combination of predicted mutations obtained by the in silico approach, without any crystal structure, promoted a 450-fold increase in affinity to DENV4, while affinity to DENV1–3 remained unchanged [157]. The structure-guided approach was then applied introducing other mutations to improve affinity and produce broad mAb neutralization [158]. Cynomolgus macaques infected with DENV2 were treated with VIS513 24 h post-onset of viremia or 5 days after infection, at the peak of viremia, and the mAb abrogated the infection, while viremia was detectable during post-treatment at a lower level compared to control animals [159]. VIS513 was evaluated in two DENV-infected mouse models, AG129 for primary infection and A129 for secondary infection of maternal antibody-mediated enhanced infection. Both groups showed viral load reduction and no mortality [160]. All studies of the VIS513 mAb showed that this engineered mAb was a promising DENV therapeutic antiviral drug.

One mAb for TBEV was also engineered. The chimeric mAb ch14D5 binds with high affinity to the Far Eastern subtype, and with lower affinity to the Siberian and European subtypes. Rational design and machine-learning methods were applied to generate engineered mAb with higher affinity to the Siberian and European subtypes [166].

The development of bispecific mAbs is an alternative approach for the combination of two or more therapeutic mAbs to minimize viral escape and ADE. Bispecfic mAb for ZIKV was developed. Initially, a panel of anti-ZIKV mAbs was identified from four ZIKV-infected patients with two of them DENV-naïve, and it was then grouped by affinity of B cells to the ZIKV NS1 and E protein and also by the neutralizing potency to ZIKV and DENV DIII of the E protein or quaternary epitope displayed on the infectious virions [47]. The epitope of the ZKA190 mAb isolated from this panel was further investigated, and it was located in the E protein in the DI-DIII linker and the lateral ridge region of DIII, a conserved epitope [162]. In a mouse model, ZIK190 was capable of delaying morbidity and mortality in a prophylactic approach, and its LALA variant did not show ADE. In the therapeutic approach, >80% survival rates were achieved, as well as a reduction in morbidity [162]. Despite that, a ZIKV escape mutant was detected in vivo, which completely abrogated ZIK190 neutralization activity [162]. A bispecific mAb was developed to circumvent this problem, and ZIK185 mAb, targeting DII of the E protein, was chosen to combine with ZIK190 [162]. Escape mutants also emerged from ZIK185 mAb, indicating that this phenomenon occurs in mAbs binding to distinct epitopes [162]. A tetravalent symmetric format Fabs-in-tandem-Ig (FIT-Ig) was constructed with ZIK190 and ZIK185 Fabs, combined with the engineered Fc backbone with the LALA mutation, and FIT-1 bispecific antibody was produced [162]. In a mouse model, FIT-1 provided protection against lethal infection in all cases, with the detection of no viral load, and nonetheless, no escape mutant was detected in vitro or in vivo [162].

Finally, Table 6 summarizes the therapeutic efficacy of mAbs developed for arboviruses using various animal models.

## 5. Conclusions

The lessons learned with the COVID-19 pandemic showed that it is important to have continuous support to develop mAbs for emerging diseases, and to be prepared to respond faster when new threads appear. Passive immunotherapy should be an effective alternative for the treatment of infectious diseases, especially for immunocompromised patients and individuals for whom the vaccination is not indicated.

Neutralizing mAbs with therapeutic potential have been developed to fight arboviruses using advanced mAb discovery technologies. Many extensive B-cell studies allowed the in-depth analysis and understanding of the immune response elicited by infected donors during the acute-phase, post-infection period, secondary infection phase and other times.

Studies related to the discovery of the protective arbovirus mAbs are also important for the rational vaccine design giving complementary information to obtain potent vaccines with better protective features.

There are many challenges to obtaining mAbs for arbovirus diseases with desired features, such as highly potent and broadly neutralizing agents. Engineering approaches could improve mAb characteristics and lead to this objective. The technology is always advancing, and novel techniques will be introduced as soon as proof of concept is shown to them, which will allow the selection of mAbs with improved and refined features.

## Figures and Tables

**Table 1:** Therapeutic mAbs for dengue disease with neutralizing activity to four serotypes.

mAb	Epitope	Technology	Cross-NEUT	Cross-REACT	Format	ADE	Ref
4E11	E protein (DIII)	hybridoma	ND	ND	Fab	ND	[75]
2A10G6	E protein (FL)	hybridoma	YFV, WNV	JEV, TBEV	IgG1	ND	[76,77]
3G9	E protein (FL)	hybridoma (B cells from secondary infection patient)	JEV, WNV, ZIKV	ND	IgG1	Yes	[78]
SIgN-3C	Inter-dimer interface of E protein	plasmablast sorting	ZIKV	ZIKV	IgG1	Yes	[79,80]
d448	Interface of E and M protein (DII)	B-cell sorting (immunized Rhesus macaque)/chimerization	YFV, WNV	No	IgG1	ND	[81]

FL: fusion loop; ND: not determined; NEUT: neutralization; REACT: reactivity; Ref: references.

**Table 2:** Therapeutic neutralizing mAbs for ZIKV developed for Zika infection.

mAb	Epitope	Technology	Cross-NEUT	Cross-REACT	Format	ADE	Ref
ZIKV-117	E protein (inter-dimer interface)	hybridoma (human B cells)	No	No	IgG1	ND	[104,105]
DH1017.IgM	E protein (inter-dimer interface, DI and DII)	memory B-cell sorting	No	No	IgM	No	[46]
Z004 + Z021	E protein (lateral ridge of DIII/DI-DIII hinge)	memory B-cell sorting	DENV1	DENV1	IgG1	Yes	[101,106]
Z23	E protein (DIII)	memory B-cell sorting	No	No	IgG1	ND	[107]
Z3L1	E protein (DI-DII)	memory B-cell sorting	No	No	IgG1	ND	[107]
ED1-B10	E protein dimer	plasmablast sorting (dengue convalescent patient)	DENV 1–3	ND	IgG	Yes	[85,108,109]

ND: not determined; NEUT: neutralization; REACT: reactivity; Ref: references.

**Table 3:** Therapeutic neutralizing mAbs for CHIKV infection.

mAb	Epitope	Technology	NEUT	Cross-REACT	Format	Ref
CHK-152 + CHK-166	E2/E1 protein	Hybridoma	CHIKV Asian, ECSA, WA	ND	IgG2	[114,120]
4N12	E2 protein	hybridoma (human B cells)	CHIKV Asian, ECSA, WA	ND	IgG2	[115]
SVIR023	E2 protein	hybridoma (human B cells)	CHIKV Asian, ECSA, WA	ND	IgG1	[121]
SVIR001	E2 protein	CDR grafting humanization	CHIKV ECSA	ND	IgG1	[122]
IM-CKV063	E2 protein (DA)	phage display/immune library	CHIKV pseudovirus	SFV	IgG1	[123]
CC3	E1 protein	phage display	CHIKV strain 181/25	ND	VHH	[124]
DC2.271B	E2 protein	B-cell sorting	CHIKV Asian, ECSA	ND	IgG1	[116]

CHIKV Asian: Asian genotype; CHIKV ECSA: East/Central/South African genotype; CHIKV WA: West African genotype; DA: Domain A of E2 protein; ND: not determined; NEUT: neutralization; REACT: reactivity; Ref: references; SFV: Semliki Forest virus; VHH: variable heavy domain.

**Table 4:** Therapeutic neutralizing mAbs for WNV.

mAb	Epitope	Technology	Neutralization	Format	Ref
MGAWN1	E protein (DIII)	hybridoma/CDR grafting	WNV I-II	IgG1	[131,132,133,134,135,136]
WNV-86	E protein (DII)	hybridoma (human B cells)	WNV I	IgG	[137]
CR4354	E protein (DIII)	phage display/immune library	WNV I	IgG1	[138,139,140]
WN_83	E protein (DIII)	single B-cell sorting	WNV I	IgG1	[141]
MIT89	E protein	single B-cell sorting	WNV I	IgG1	[55]

**Table 5:** MAbs for arboviruses obtained by next-generation strategies.

mAb	Arbovirus	Epitope	Engineering Strategy	Engeneering Purpose	Format	Ref
VIS513	DENV	E protein (DIII)	Humanization of 4E11 by in silico approaches (predicted and guided mutations)	Enhance neutralization and achieve broad neutralization	IgG1	[157,158,159,160]
SIgN-3C	DENV	Inter-dimer interface of E protein	LALA mutation	Abrogate ADE	IgG1	[79]
3G9	DENV	E protein (fusion loop)	LALA mutation or N265A or N297A	Abrogate ADE	IgG1	[78]
ED1-B10	DENV/ ZIKV	E protein dimer	LALA mutation	Abrogate ADE	IgG	[108]
Z021 + Z004	ZIKV	E protein/E protein	Fc engineering by GRLR/LS modification	Abrogate ADE	IgG1 (cocktail)	[106]
SMZAb1 + SMZAb2 + SMZAb5	ZIKV	E protein (DIII/DII/DIII)	LALA mutation	Abrogate ADE	IgG1 (cocktail)	[161]
FIT-1 (ZKA190 + ZKA185)	ZIKV	E protein (DI-DIII linker and DIII/DII)	tetravalent symmetric format Fabs-in-tandem-Ig (FIT-Ig) for bispecific construction and LALA mutation	Abrogate ADE, enhance neutralization and prevent escape mutants	Bispecific mAb	[162]

**Table 6:** In vivo therapeutic efficacy of mAbs for arboviruses.

mAb	Arbovirus	Therapeutic Efficacy
3G9-N297A	DENV	Immunocompetent BALB/c mice were infected with DENV2, and mAb treatment reduced viremia [78].
SIgN-3C-LALA	DENV	mAb treatment decreased the viremia of four serotypes in infected-mice [79].
VIS513	DENV	mAb was administered after DENV2 infection in cynomolgus macaques, and viremia was reduced [159]. Mouse model for primary and secondary infection received mAb treatment, and viremia decreased [160].
ZIKV-117	ZIKV	ZIKV-infected mouse dams were treated with mAb and the viral burden was decreased in mother, placenta and fetal tissues, with fetal demise also reduced [104].
DH1017.IgM	ZIKV	ZIKV-infected mice were treated with mAb, and viremia was reduced [46].
Z004 + Z021	ZIKV	Macaques were ZIKV-challenged and received two mAbs, and low level of viremia was observed [106].
EDE1-B10	ZIKV	mAb was given to ZIKV-infected mice, and RNA level was reduced in immune-privileged sites (serum, brain, epididymis, eye). In pregnant mice, infection and injury to the placenta and fetus were prevented [108]. ZIKV-infected Rhesus monkeys were treated with mAb, and viremia decreased [109].
SMZAb1 + SMZAb2 + SMZAb5	ZIKV	mAb cocktail was administered to Rhesus monkeys before ZIKV infection, and viral replication was prevented [161]. In pregnant macaques ZIKV-infected, the treatment was effective in clearing the virus, but viral RNA was present in amniotic fluid; treatment failed to prevent fetal demise [165].
FIT-1	ZIKV	Mice received FIT-1 mAb (ZKA190+ ZKA185) before ZIKV-challenge and viral titers were abrogated [162].
CHK-152 + CHK-166	CHIKV	A combination of mAbs was given to CHIKV-infected mice, and they were protected [114]. CHIKV-infected Rhesus macaques were treated with two mAbs, and viral burden was low [120].
SVIR023	CHIKV	CHIKV-challenged mice were treated with mAb and showed reduced virus titer and resistance to secondary infection [121].
SVIR001	CHIKV	mAb was administered to CHIKV-infected mice, and viremia was reduced. In Rhesus macaques, viremia was eliminated, and CHIKV-associated inflammatory diseases were decreased [122].
hE16 (MGAWN1)	WNV	WNV-infected hamsters received mAb, and the neurological disease was ameliorated after viral replication [134].
WNV-86	WNV	Mice were WNV-challenged and treated with mAb, and reduced viral burden was observed in the spinal cord and brain [137].
chFVN145	TBEV	TBEV-infected mice received mAb, and efficacy was dose-dependent [154].
T025	TBEV	Mice were TBEV-infected and treated with mAb, and early treatment was effective [156].

## Data Availability

Not applicable.
